# An examination of the associations between depressive symptoms, perceived parental discipline, alcohol use, and drinking-related consequences during the first year of college: A moderated mediation model

**DOI:** 10.1016/j.jadr.2023.100603

**Published:** 2023-06-03

**Authors:** Reed M. Morgan, Bradley M. Trager, Sarah C. Boyle, Joseph W. LaBrie

**Affiliations:** a Department of Psychological Science, Loyola Marymount University, 1 LMU Drive Suite 4700, Los Angeles, CA 90045, United States

**Keywords:** Depression, Alcohol use, Drinking consequences, Parent influence, Discipline, College

## Abstract

**Background:**

Depression is prevalent among adolescents and young adults and is associated with experiencing increased negative alcohol-related consequences; thus, it is imperative to identify malleable protective factors for alcohol risks in young adults experiencing elevated depressive symptoms. The current study longitudinally explored the effects of perceived parental alcohol-related discipline on the relationship between depressive symptoms, alcohol use, and negative drinking consequences during the transition into college.

**Methods:**

Incoming college students (*N* = 272, 63.2% female) completed web-based surveys before (July, T1) and after (October, T2) the transition into college and reported depressive symptoms, perceived alcohol-related discipline, alcohol use, and consequences of drinking experienced in the past 30 days.

**Results:**

The moderated mediation model revealed that at above average perceptions of alcohol-related discipline, depressive symptoms were negatively associated with alcohol use, which in turn was associated with experiencing fewer negative consequences of drinking.

**Limitations:**

The current study did not measure a diagnosis of major depressive disorder, and therefore our results may differ among clinical populations. In addition, we did not measure other parenting constructs shown to protect students with elevated levels of depressive symptoms from experiencing consequences (i.e., monitoring).

**Conclusions:**

The present findings suggest perceptions of parental alcohol-related discipline measured here (e.g., having a privilege taken away, being scolded or grounded) can be protective against alcohol risks among college students experiencing above average depressive symptoms. Parent-based alcohol interventions administered prior to matriculation should encourage parents of depressed students to clearly communicate consequences for drinking to their child.

## 1. Introduction

Depression is a significant issue for late adolescents and young adults, with an estimated 5.6 million 18–25-year-olds in the U.S. (17%) experiencing a major depressive episode in the past year (Substance Abuse and Mental Health Services Administration [SAMHSA], 2021). From 2010 to 2018, the number of young adults with major depressive disorder (MDD) increased approximately 58% and the economic burden caused by MDD among all adults rose 38% to $326.2 billion in the U.S. alone (Greenberg et al., 2021). Depression is associated with a plethora of negative outcomes among young adults, including risky drinking and alcohol-related negative consequences (SAMHSA, 2021), discontinued college enrollment (Arria et al., 2013), self-harm (Moran et al., 2012), and suicidal behavior (Soto-Sanz et al., 2019). When considering these potential harms along with the fact that only about 58% of 18–25-year-olds in the U.S. who experienced a major depressive episode in the past year received treatment for depression (SAMHSA, 2021), more must be done to reduce the negative consequences of depression among young adults.

With respect to comorbid alcohol problems, college students are at particularly high risk for both depression (Ibrahim et al., 2013) and risky drinking (Schulenberg et al., 2021; H. R. White et al., 2006), and the transition to college is a period associated with increases in depression (Iyer et al., 2009), drinking, and consequences (Riordan and Carey, 2019), as well as the development of longstanding patterns of risky drinking (Derefinko et al., 2016). While the literature is inconsistent as to whether a direct relationship between depression and alcohol use exists among college students (Geisner et al., 2018; Kenney et al., 2018; Weitzman, 2004), there is a reliable positive association between depressive symptoms and negative consequences of alcohol use (Acuff et al., 2018; Kenney et al., 2015; Rosenthal et al., 2018). This suggests that while depressed individuals do not necessarily drink more than their mentally well peers, they are more likely to experience serious and harmful outcomes of drinking such as blacking out, throwing up, or declining academic performance. Consequences have been shown to predict depression as well (Rosenthal et al., 2018), which is problematic as depression and alcohol consequences may create a feedback loop in which depression results in experiencing increased negative consequences of alcohol, and experiencing those consequences then worsens depressive symptoms. The relationship between depression and consequences may be due in part to cognitive and motivational factors such as drinking to cope (e.g., Bravo and Pearson, 2017; Bravo et al., 2018; Kenney et al., 2018) and the use of protective behavioral strategies (Martens et al., 2008), both of which have been shown to mediate the effects of depression on alcohol consequences. Specifically, depression was positively associated with drinking to cope (Bravo and Pearson, 2017; Bravo et al., 2018; Kenney et al., 2018) and protective behavioral strategies were negatively associated (Martens et al., 2008); drinking to cope was in turn positively associated with consequences. Considering that drinking behavior only accounts for approximately half the variance in alcohol consequences (e.g., Larimer et al., 2004; Mallett et al., 2011; Turner et al., 2000) and given the reciprocal relationship between depression and negative consequences of alcohol, it is crucial to identify factors that contribute to, or are protective against, alcohol-related consequences among college students experiencing symptoms of depression.

One reliable mechanism for alcohol-related behavior change among students during the transition to college is parental influence (e.g., Abar et al., 2009; Napper, 2019; Varvil-Weld et al., 2014; Wood et al., 2004). Parents’ pre-matriculation attitudes toward drinking and rules about alcohol use have reliably been shown to influence their student’s drinking and consequences during the first year of college (Turrisi and Ray, 2010) and beyond (Abar et al., 2014; Walls et al., 2009). Furthermore, there is evidence that parenting behaviors affect relationships between symptoms of depression and alcohol-related consequences among first-year college students: Geisner et al. (2018) found that parental monitoring significantly moderated the association between symptoms of depression and negative alcohol-related consequences. Among those high in depressive symptoms, consequences decreased as parental monitoring increased. Therefore, other parenting variables associated with decreases in drinking might also moderate the relationship between symptoms of depression and negative consequences.

Adolescents’ perceptions that their parents would discipline them if they were caught drinking, for example by taking away belongings or privileges, scolding or yelling at them, or grounding them, can be a protective factor for adolescent drinking (Cox et al., 2018; Foley et al., 2004; Reboussin et al., 2006; Song et al., 2012) and consequences (Song et al., 2012). For instance, Reboussin et al. (2006) found that adolescents’ perceptions that their parents would discipline them for drinking were associated with a decreased likelihood of engaging in high-risk drinking. Furthermore, adolescents who perceived that their parents would yell/punish them if they were caught drinking (vs. those who did not) reported fewer drinks consumed on their last drinking occasion and were less likely to have had a drink or been drunk in the past 30 days, engaged in heavy episodic drinking in the past two weeks, driven under the influence or ridden in a car with someone who had been drinking, and experienced nonviolent negative consequences of alcohol use (Foley et al., 2004; Song et al., 2012). More recently, these perceptions have been found to be negatively correlated with ever having a full drink or heavy drinking episode (Cox et al., 2018). Importantly, these findings are specific to the kinds of discipline measured by these studies and do not suggest that any form of discipline would be effective; certain punishments, particularly corporal punishment, are not recommended because they are associated with negative outcomes such as aggression, criminal and antisocial behavior, and decreased mental health (e.g., Gershoff, 2002). Together, these findings suggest that perceived parental alcohol-related discipline is protective against general and risky drinking during adolescence and its influence might extend through the transition into college and beyond.

In addition, perceptions of discipline may be especially protective for individuals with depressive symptoms. Among both clinical and non-clinical populations, being depressed has been shown to associate with increased sensitivity to punishment (Hevey et al., 2017; Kim et al., 2021; Kong et al., 2022). Furthermore, depressed adults exhibit less risk-taking behavior when compared to non-depressed controls (Smoski et al., 2008). Taken together, it is plausible (but remains untested) that perceived alcohol-related discipline may moderate the relationship between depression and drinking. If this is the case, targeting parenting behaviors that lead to perceptions of discipline may reduce alcohol-related risks among college students experiencing depressive symptoms.

### 1.1. Current study

**Fig. 1.**
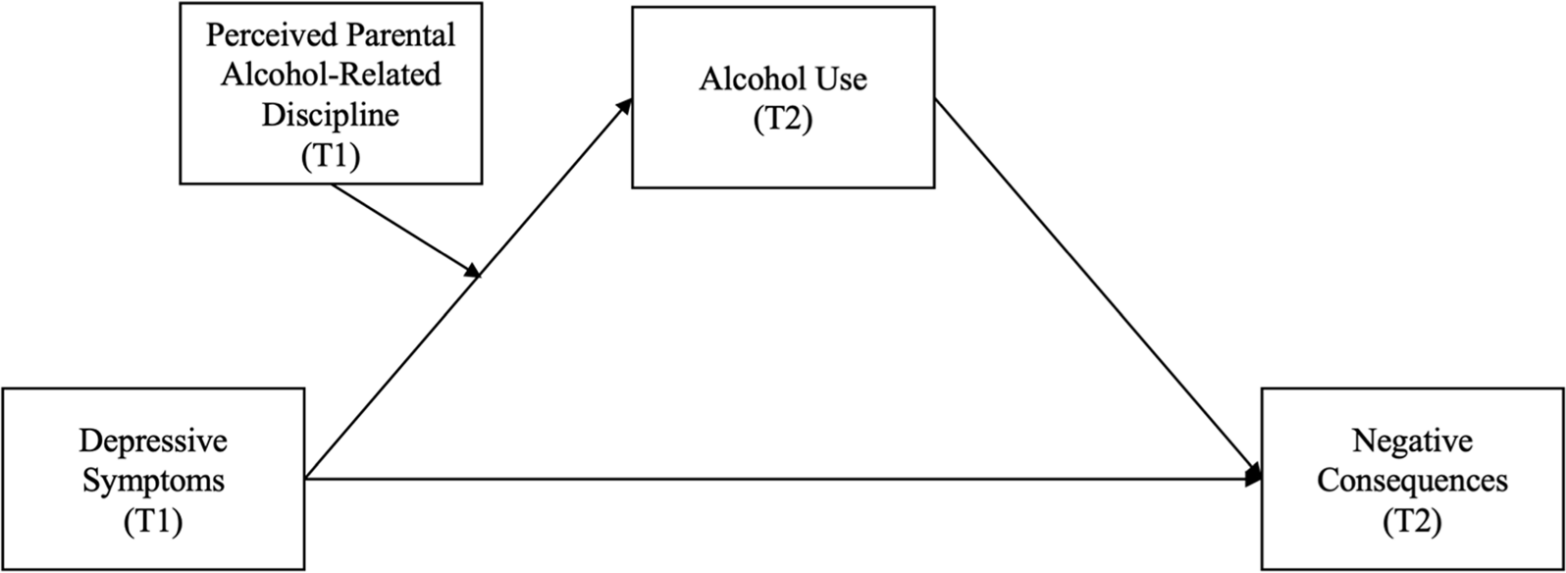
Conceptual first-stage moderated mediation model. Included in the model but not pictured are the following T1 covariates: alcohol use, negative consequences, parental alcohol use, birth sex, race, and ethnicity.

As illustrated in Fig. 1, the current study used a longitudinal design to investigate whether depressive symptoms during the transition to college indirectly influenced negative alcohol consequences via drinking, and whether perceived alcohol-related discipline moderated the relationship between depressive symptoms and drinking. Data for this study were collected before and after matriculation, during the same period in which parent-based interventions (PBIs) (e.g., LaBrie et al., 2016; Turrisi et al., 2001) have been found to be most effective in this population (i.e., the pre-matriculation summer months; Turrisi et al., 2013). Therefore, findings can be used to inform PBIs about any potential benefits or risks alcohol-related discipline might have on alcohol use in general and among depressed incoming first-year students. Based on the literature described thus far, we hypothesized that perceived alcohol-related discipline would moderate the relationship between depressive symptoms and alcohol use, which in turn would predict drinking-related consequences. Specifically, we predicted that among those with greater perceptions of parental alcohol-related discipline, increased symptoms of depression would be associated with less drinking, which in turn should predict experiencing fewer negative consequences.

## 2. Method

### 2.1. Recruitment and procedures

The sample consisted of incoming first-year college students from a private, mid-sized university on the West Coast who were recruited to participate in a RCT evaluating the effectiveness of a parent-based intervention.^1^ Participants were recruited via email invitation to complete web-based surveys pre- (July 2021; T1) and post-matriculation (October 2021; T2). Participants received a $25 electronic gift card per survey. Of those who participated at T1 (*N* = 391), approximately 72% (*n* = 281) completed all follow-up measures, and, of those, nine participants were dropped because they reported inconsistent responses to drinking items between assessments (i.e., drinkers at T1 reported never having tried alcohol at T2). Participants included in the final set of analyses (*n* = 272) were higher on T1 depressive symptoms (*t*(184.47) = 2.24, *p* = .026) compared to those who could not be included because they did not complete all study variables/were dropped from subsequent analyses (*n* = 119). No other significant differences were detected between these two groups. All study procedures were approved by the university’s IRB.

### 2.2. Participants

Participants (*N* = 272) were 17 to 20 years of age (*M*_*age*_ = 17.89, *SD*_*age*_ = 0.42) and the majority were born female (i.e., birth sex; 63.2% female, 36.8% male) and identified as female (i.e., gender identity; 63.2% female; 36.4% male, 0.4% other). The racial and ethnic makeup was diverse and in line with the university’s undergraduate student body: 49.6% White; 20.6% multiracial; 14.0% Black or African American; 12.5% Asian; 0.4% American Indian or Alaska Native; and 0.4% Native Hawaiian or Other Pacific Islander (2.6% missing); 25.4% were Hispanic/Latino. Students were primarily heterosexual/straight (81.3%; 8.5% bisexual, 5.1% unsure, 2.9% queer, 2.2% gay/lesbian).

### 2.3. Measures

Descriptive statistics for all study variables are included in Table 1.

#### 2.3.1. Depressive symptoms

The depression subscale of the 21-item Depression, Anxiety, and Stress Scale (DASS-21; Antony et al., 1998; Lovibond and Lovibond, 1995) was administered at T1 to measure depressive symptoms in the past week. Participants were asked to indicate how often each statement applied to them on a 4-point scale from “Did not apply to me at all” (0) to “Applied to me very much, or most of the time” (3). Responses to each of the seven items were summed and multiplied by two (α = .89), in keeping with the original scoring of the measure and to make final scores comparable to those of the longer 42-item version of the DASS (Lovibond and Lovibond, 1995).

#### 2.3.2. Alcohol use index

To assess alcohol consumption, participants were asked to report their typical weekly drinking, drinks per occasion on their typical drinking occasion, drinks per occasion on their heaviest drinking occasion, and frequency of heavy episodic drinking (HED), all within the past 30 days. Typical weekly drinking was assessed using the Daily Drinking Questionnaire (Collins et al., 1985). Participants were asked to indicate how many drinks they consumed on each day of a typical week, and answers were then summed to create a measure of weekly drinking (T1: *M* = 1.66, *SD* = 3.45; T2: *M* = 3.19, *SD* = 4.53). To assess drinking on students’ typical occasion, they were asked, “During the past 30 days, on average, how many drinks did you have each time you drank?” with answers ranging from 0 to 25+ (T1: *M* = 1.01, *SD* = 1.59; T2: *M* = 1.73, *SD* = 2.18). To assess peak drinking, participants were asked, “During the past 30 days, what is the maximum number of drinks you drank during a single drinking occasion?” with answers ranging from 0 to 25+ (T1: *M* = 1.73, *SD* = 2.71; T2: *M* = 2.50, *SD* = 3.04). To assess frequency of engaging in HED, participants were asked “During the past 30 days, how many times did you have [four/five] or more drinks within a 2-hour period?” for females and males, respectively, with answers ranging from 0 to 7+ (T1: *M* = 0.31, *SD* = 0.79; T2: *M* = 0.65, *SD* = 1.29) (National Institute on Alcohol Abuse and Alcoholism, n.d.). Outlier adjustment was performed for typical weekly drinking (T1) and frequency of HED (T1 and T2) because skews and kurtoses were greater than 2 and 5, respectively (Tabachnick and Fidell, 2019). Specifically, outlying values were winsorized to 3.29 *SD*s above the mean. To comprehensively assess participants’ drinking using a single variable, these four measures of alcohol use were then standardized and averaged to create an index of alcohol use at each timepoint. Reliability was excellent at T1 (α = .93) and T2 (α = .95).

#### 2.3.3. Negative drinking consequences

Consequences of alcohol use were assessed using 11 items from the Brief Young Adult Alcohol Consequences Questionnaire (BYAACQ; Kahler et al., 2005) and three items adapted from the Young Adult Alcohol Problems Severity Test (YAAPST; Hurlbut and Sher, 1992). The included items were selected to be consequences most appropriate for incoming first-year students that indicate physical, academic, social, and sexual consequences of drinking (see LaBrie et al., 2016), and achieving this by combining items from these two measures is common (e.g., Lee et al., 2014; Reavy et al., 2016; Trager et al., 2019). Participants indicated if and how many times they experienced each consequence in the past 30 days on a 6-point scale from “No, never” (0) to “7+ times” (5). Examples of given items include “I have passed out from drinking” and “I have neglected my obligations to work, family, or school because of drinking.” Responses were dichotomized to indicate whether participants reported that they did (1) or did not (0) experience each consequence and dichotomized responses were then summed at T1 and T2. Outlying values were winsorized to 3.29 *SD*s from the mean at both timepoints.

#### 2.3.4. Perceived parental alcohol-related discipline

Perceived alcohol-related discipline was assessed at T1 using four items adapted from Chassin et al. (1998). This measure, which originally assessed parental discipline for smoking, has previously successfully been adapted for drinking (Cox et al., 2018). Participants were asked, if their parent(s) found out that they were drinking, how likely it would be that their parents would (1) “Take away something from you, like your car or allowance”, (2) “Ground you”, (3) “Take away a privilege, like watching TV or using a cell phone”, and (4) “Scold/yell at you.” Each item was given on a 5-point scale from “Definitely not” (0) to “Definitely” (4). Responses were summed to create a composite variable of perceived alcohol-related discipline (α = .93).

#### 2.3.5. Parental alcohol use

Five items taken from Abar et al. (2009) were given to assess parental alcohol use: “While growing up, how often…” (1) “did you see your mother drink alcohol?”, (2) “did you see your father drink alcohol?”, (3) “was alcohol on the dinner table during family meals?”, (4) “did you see your mother drunk from alcohol?”, and (5) “did you see your father drunk from alcohol?” For each item, answer options ranged from “Not at all” (0) to “Everyday” (5). Items were averaged to create a composite variable of parental alcohol use (α = .81).

### 2.4. Analytic plan

PROCESS Model 7 (Hayes, 2022) was used to test the first-stage moderated mediation model illustrated in Fig. 1. In the model, follow-up alcohol use index (mediator) was regressed onto baseline depressive symptoms, perceived alcohol-related discipline, depressive symptoms*perceived alcohol-related discipline, and baseline covariates (alcohol use index, consequences, parental alcohol use, birth sex, race, and ethnicity),^2^ and follow-up consequences (outcome) was regressed onto the mediator, baseline depressive symptoms, and covariates. Predictor variables (i.e., depressive symptoms and perceived alcohol-related discipline) were mean-centered by the PROCESS macro as part of the analysis. Bootstrapped 95% asymmetric confidence intervals (CIs) were computed (*N* = 10,000 samples; Hayes, 2022; MacKinnon et al., 2002), and model effects were considered significant at *p* < .05 when CIs excluded zero.

## 3. Results

Correlations between variables included in the moderated mediation model are provided in Table 1. As expected, T1 depressive symptoms were positively correlated with T2 consequences (*r* = .19, *p* = .002), but they were not significantly correlated with T2 alcohol use index (*r* = .01, *p* = .828). Also expected, T2 alcohol use index was highly positively correlated with T2 consequences (*r* = .63, *p* < .001), and T1 perceived alcohol-related discipline was negatively correlated with T2 alcohol use index (*r* = −.28, *p* < .001) and T2 consequences (*r* = −.20, *p* = .001).

**Fig. 2.**
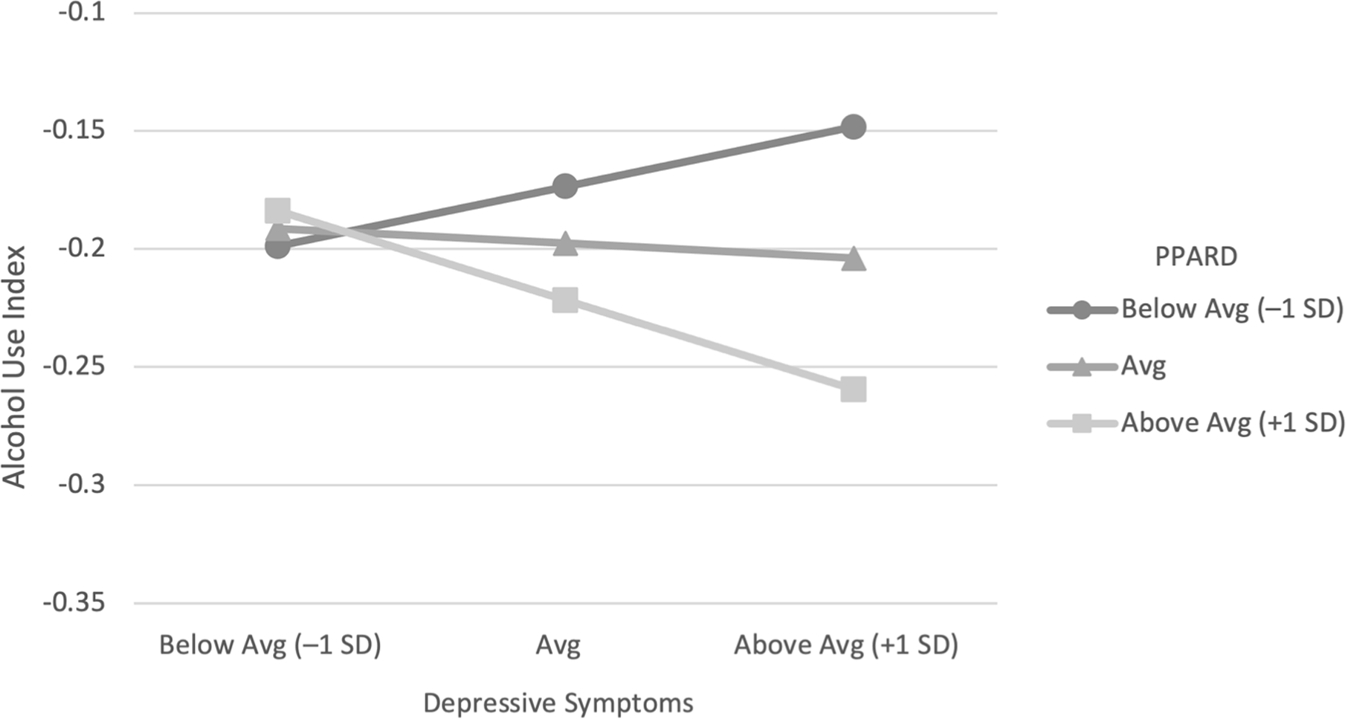
Interaction between depressive symptoms and perceived parental alcohol-related discipline (PPARD) predicting alcohol use index.

Table 2 includes results from the moderated mediation model. Results revealed that T1 perceptions of alcohol-related discipline moderated the effects of T1 depressive symptoms on T2 alcohol use index (*b* = −0.002, *SE* = 0.001, 95% CI [−0.004, −0.0004]; see Fig. 2), and T2 alcohol use index predicted T2 consequences (*b* = 1.158, *SE* = 0.21, 95% CI [0.79, 1.62]). These results suggest that there was significant moderated mediation (Index = −0.003, *SE* = 0.001, 95% CI [−0.01, −0.001]); examination of the conditional indirect effects revealed moderated mediation occurred at high levels of perceived alcohol-related discipline. The indirect coefficient for above average levels of perceived alcohol-related discipline was significantly less than what was observed at the average and below average levels. The indirect effect for individuals reporting average perceived alcohol-related discipline was also significantly less than what was observed at low levels. These results provide evidence of first-stage moderated mediation in which the indirect negative relationship between depressive symptoms and alcohol consequences via alcohol use index were strongest among students with high perceptions of parental alcohol-related discipline relative to those with average or below-average perceptions.

## 4. Discussion

This study found that the relationship between depressive symptoms and negative consequences of drinking is mediated by alcohol use, but only when perceived parental discipline is high. At high levels of perceived parental discipline, increases in depressive symptoms led to decreases in alcohol use, which in turn predicted fewer negative consequences of drinking. These findings suggest that at higher levels of perceived parental alcohol-related discipline, these perceptions become more protective against alcohol risk as depressive symptoms increase. This study extends previous research on the benefits of perceived alcohol-related discipline (Cox et al., 2018; Foley et al., 2004; Reboussin et al., 2006; Song et al., 2012) by demonstrating that (a) the protective effects of these perceptions on risky drinking extend to the transition to college, and (b) these perceptions may be more effective at reducing alcohol-related risk among individuals who report more depressive symptoms. Results from this study also support previous research that suggests depressive symptoms are more reliably associated with alcohol consequences than alcohol consumption (e.g., Martens et al., 2008). Findings from the moderated mediation model suggest that other variables affect the relationship between depression and drinking. For instance, we did not find a bivariate relationship between depressive symptoms and alcohol use, which is consistent with previous research (e.g., Geisner et al., 2018). However, when taking perceived alcohol-related discipline into account, we found that students who had higher perceptions of alcohol-related discipline and were more depressed drank less than those who were less depressed. Taken together, findings from this study offer support for the protective effects of perceived non-physical alcohol-related discipline among individuals who are more depressed and suggest that examining moderators might clarify the relationship between depressive symptoms and alcohol use.

Our findings indicated that moderated mediation only occurred at above average levels of perceived alcohol-related discipline. While this may be because low or average perceptions of alcohol-related discipline are not salient enough or a strong enough deterrent against drinking to affect students’ behavior, it was likely an artifact of our sample. Participants reported symptoms in the “normal” range on average (i.e., a score of 0–9; Lovibond and Lovibond, 1995); low endorsement of depressive symptoms likely made it difficult to detect effects among individuals at average or below average perceptions of alcohol-related discipline, in which we would already expect these effects to be weaker. Notably, students who completed all survey timepoints and were included in our analytic sample had higher depressive symptoms than those who were excluded, which possibly aided our ability to detect effects. However, the effect size of this difference was small (*d* = 0.26) and likely had little impact on our results. To better understand the relationships between our study variables, replication studies using larger samples endorsing higher levels of depressive symptoms are needed.

The current findings are consistent with research using both clinical and non-clinical populations which has shown that depression is associated with increased sensitivity to punishment (Hevey et al., 2017; Kim et al., 2021) and decreased risk-taking behavior (Smoski et al., 2008). Although bivariate correlations between depressive symptoms and drinking were not significant, our moderated mediation model revealed that among those who experienced above average perceptions of alcohol-related discipline, those with more depressive symptoms demonstrated decreased risk-taking behavior by consuming less alcohol than their less-depressed peers. In this context, it is likely that students experiencing elevated depressive symptoms who believe that their parents would discipline them for drinking might be particularly motivated to avoid discipline, and therefore are more likely to abstain from drinking so as to not get disciplined for doing so. However, one important distinction between past studies and the present one is that past research explored behavior following an experienced instance of discipline, not perceived discipline as was measured in the current study. While a similar pattern of results to those observed here is likely since students who have been disciplined are likely to have greater perceptions of alcohol-related discipline, future research should explore the effects of having been disciplined by a parent for drinking in the past as this may help to better explain the relationship between depression, perceptions of discipline, drinking, and consequences among incoming college students.

This study also expands the literature that suggests certain parenting behaviors can be protective against drinking risk among individuals experiencing elevated symptoms of depression (Geisner et al., 2018). Increasing parental monitoring or perceptions of discipline are two simple and effective ways to reduce negative drinking-related consequences of depression in incoming college students that can be accomplished by any parent. It is plausible that employing these two strategies together may further decrease drinking risk compared to using either in isolation—for instance, if parents monitor their adolescent children with the intent of preventing them from drinking but do not discipline them if they drink, their children may learn that their parent’s monitoring is superficial and its impact on adolescents’ behavior may lessen over time. Since parents can play an important role in protecting their young adult children from risks associated with the transition to college, such as alcohol use and even depression, they should be encouraged to capitalize on this positive influence by monitoring their children and communicating clear consequences for drinking.

## 5. Implications

Our findings have implications for intervention programs designed to reduce risks associated with depression and alcohol use among adolescents. Increasing students’ perceptions of alcohol-related discipline may help to reduce drinking risk during the first year of college among students with high levels of depressive symptoms. To accomplish this, information about parental discipline can be included in PBIs delivered pre-college matriculation (e.g., LaBrie et al., 2016; Turrisi et al., 2001). Specifically, parents should be informed that clearly communicating with their child about how they will respond to their son or daughter’s drinking may be protective against alcohol risk, especially if their student is depressed. This information could be given alongside other similar recommendations such as setting clear expectations with their adolescent that they are not permitted to drink, as this has been shown to be protective against alcohol use (LaBrie et al., 2015). It is important to note that our findings may not extend to forms of punishment not represented in our measure of perceived discipline. For instance, the use of corporal punishment is reliably associated with negative outcomes (e.g., Gershoff, 2002) and should not be recommended to parents. Parents should also be made aware that setting and enforcing consequences for drinking should be accompanied with parental warmth and open communication or else they run the risk of these perceptions backfiring and increasing risks for depression and risky drinking (Patock-Peckham and Morgan-Lopez, 2007, 2009). Authoritative parenting, characterized by parental warmth and nurturing coupled with the setting of firm boundaries (Baumrind, 1971), has been shown to be indirectly associated with decreased depression and alcohol consequences (Patock-Peckham and Morgan-Lopez, 2009) while authoritarian parenting, marked by emotional coldness and the strict enforcement of punitive measures (Baumrind, 1971), has been shown to indirectly associate positively with these risks (Patock-Peckham and Morgan-Lopez, 2007, 2009). Examining parental warmth and discipline together in future research may help us better understand how parents of young adults experiencing symptoms of depression can protect their children from alcohol consequences.

### 5.1. Limitations and future directions

The current study had some limitations that should be addressed in future research. While the depression subscale of the DASS-21 is a valid measure of depressive symptoms in both clinical and non-clinical samples (e.g., Antony et al., 1998), it does not represent a clinical diagnosis for MDD and thus our findings may not extend to clinical populations. It is plausible that the observed effects may be stronger in a clinical sample as sensitivity to punishment can be higher among individuals diagnosed with MDD compared to a demographically similar sample experiencing symptoms of depression considered to be in the normal range (Hevey et al., 2017). Similarly, the current sample consisted of low-risk drinkers, which may affect our findings’ generalizability to heavier drinking students. Since we were able to detect effects despite participants’ low drinking, utilizing more risky samples are likely to lead to stronger effects, yet future research is required to determine if perceived alcohol-related discipline is protective for heavy drinking young adults. Third, we measured depressive symptoms over the past week which means participants’ reports may have been affected by situational differences that did not reflect their typical mood or relate to their drinking behavior months later. While this window of time was chosen because it is the original timeframe given by the DASS-21 (Lovibond and Lovibond, 1995), future research should consider measuring depressive symptoms over a longer period of time (e.g., two weeks to match the DSM-V criteria for MDD). We also did not measure and were unable to control for other parenting variables found to be protective against alcohol risk among individuals with depressed mood (i.e., monitoring; Geisner et al., 2018). Future research should investigate if the effects of perceived alcohol-related discipline are protective above and beyond the effects of other constructs, or if perhaps perceptions of alcohol-related discipline and monitoring are part of a larger cluster of parenting behaviors protective against drinking among those with elevated depressive symptoms. Next, while our sample was racially and ethnically diverse, it came from one private mid-sized university on the West Coast and therefore may not generalize to college students in different regions of the country. This study was also unable to examine gender differences given its sample size. Given reliable gender differences in both depression (Weitzman, 2004) and risky drinking among college students (A. M. White et al., 2006), future studies should explore whether the observed effects differ by gender. Finally, this study examined perceptions of alcohol-related discipline, not whether parents have disciplined them in the past or would actually discipline them if caught drinking. While students’ self-reported perceptions may be more impactful on drinking behavior than their parents’ actual behaviors (Trager et al., 2023b; Varvil-Weld et al., 2013), which suggests students’ perceptions are likely a function of things their parents have done in the past or would realistically do in the future, research should explore the impacts of actual discipline on drinking behavior among incoming college students experiencing elevated symptoms of depression.

## 6. Conclusion

The current study found that elevated perceptions that one’s parents would discipline them for alcohol use (i.e., by taking away belongings or privileges, scolding/yelling at them, or grounding them) was protective against drinking and negative alcohol-related consequences among incoming college students experiencing increased depressive symptoms. These findings suggest PBIs should encourage parents of students who are depressed to clearly communicate to their student that they are not allowed to drink and will face non-physical consequences if they do. Future research should continue to investigate cognitive factors that may protect depressed young adults from experiencing negative consequences associated with alcohol use.

## Figures and Tables

**Table 1:** Descriptive statistics and bivariate correlations between all continuous study variables.

		1	2	3	4	5	6	*M*	*SD*	*Skew*	*Kurtosis*

1	T1 Depressive symptoms	—						6.63	7.87	1.35	1.18
2	T1 PPARD	.12*	—					6.03	5.02	0.59	−0.78
3	T1 Parental alcohol use	.04	−.22***	—				1.47	0.99	0.33	−0.61
4	T1 Alcohol use index	−.01	−.28***	.34***	—			−0.05	0.86	2.00	3.91
5	T2 Alcohol use index	.01	−.28***	.32***	.72***	—		0.02	0.93	1.45	1.92
6	T1 Consequences	.12	−.29***	.33***	.70***	.67***	—	1.11	1.77	1.79	2.69
7	T2 Consequences	.19**	−.20**	.20**	.44***	.63***	.64***	1.30	2.14	2.07	4.07

PPARD = Perceived parental alcohol-related discipline.

* *p* < .05.

** *p* < .01.

*** *p* < .001.

**Table 2:** Effects of the first-stage moderated mediation model.

Predictor variables	Alcohol use index (T2) (mediator)	
	*b*(*SE*)	95% CI

Depressive symptoms (T1)	−0.001(0.01)	[−0.01, 0.01]
PPARD (T1)	−0.005(0.01)	[−0.02, 0.01]
Depressive symptoms × PPARD	**−0.002(0.001)**	**[−0.004, −0.0004]**
**Covariates**		
Alcohol use index (T1)	**0.528(0.07)**	**[0.39, 0.65]**
Negative consequences (T1)	**0.161(0.04)**	**[0.09, 0.23]**
Parental alcohol use (T1)	0.044(0.04)	[−0.04, 0.13]
Male (female = *ref*)	0.133(0.08)	[−0.03, 0.30]
White (non-White = *ref*)	−0.009(0.08)	[−0.17, 0.15]
Hispanic/Latino (non-Hispanic/Latino = *ref*)	−0.113(0.08)	[−0.27, 0.06]

Predictor variables	Negative consequences (T2)	
	*b*(*SE*)	95% CI

Depressive symptoms (T1)	**0.036(0.02)**	**[0.01, 0.07]**
Alcohol use index (T2) (mediator)	**1.158(0.21)**	**[0.79, 1.62]**
**Covariates**		
Alcohol use index (T1)	**−0.550(0.19)**	**[−0.94, −0.18]**
Negative consequences (T1)	**0.537(0.10)**	**[0.34, 0.73]**
Parental alcohol use (T1)	−0.073(0.08)	[−0.23, 0.10]
Male (female = *ref*)	−0.228(0.19)	[−0.58, 0.16]
White (non-White = *ref*)	0.077(0.19)	[−0.30, 0.45]
Hispanic/Latino (non-Hispanic/Latino = *ref*)	**0.659(0.28)**	**[0.13, 1.24]**

Conditional Indirect Effects	Negative consequences (T2)	
	*b*(*SE*)	95% CI

High PPARD (+1 *SD*)	**−0.014(0.01)**	**[−0.03, −0.001]**
Average PPARD	−0.001(0.01)	[−0.01, 0.01]
Low PPARD (−1 *SD*)	0.012(0.01)	[−0.01, 0.04]

Indirect Effect Differences	*b*(*SE*)	95% CI

High PPARD vs. Low PPARD	**−0.026(0.01)**	**[−0.05, −0.005]**
High PPARD vs. Average PPARD	**−0.013(0.01)**	**[−0.03, −0.002]**
Average PPARD vs. Low PPARD	**−0.013(0.01)**	**[−0.03, −0.002]**

PPARD = Perceived parental alcohol-related discipline. Bold denotes statistical significance (*p* < .05) based on 95% bootstrapped confidence intervals with 10,000 samples.

## Footnotes

1 Participants in this study were the 2021 cohort in Trager et al. (2023a).

2 Analyses were conducted with and without RCT condition as a covariate. Statistical significance patterns were identical when condition was controlled for, so we report the model without condition included.
